# The impact of ultra-processed foods on social development of adolescent: differences by gender and only child status

**DOI:** 10.3389/fnut.2026.1876794

**Published:** 2026-07-20

**Authors:** Chenchen Liu, Zhiwen Yang, Yuke Yang, Xinxin Lai, Yan Gao

**Affiliations:** 1 School of Medical Information Engineering, Jining Medical University, Jining, Shandong, China; 2 School of Physical Education, Shandonghity University, Jinan, Shandong, China; 3 Department of Biomedical Engineering, Faculty of Engineering, The Hong Kong Polytechnic University, Hong Kong, Hong Kong SAR, China

**Keywords:** antisocial behavior, gender, only child status, social skill, ultra-processed food

## Abstract

**Background:**

This study sought to examine the relationship between the consumption of ultra-processed foods (UPF) and both social skills (SS) and antisocial behavior (ASB) in Chinese adolescents, while also exploring the potential moderating effects of gender and only child status.

**Methods:**

A longitudinal study was conducted with 3,206 adolescents (1,510 males, 1,696 females; mean age 13.62 ± 14.09 years). The first wave of data was collected in the autumn of 2021, and the second wave was collected in the autumn of 2022 through follow-up surveys. UPF intake was assessed using the NOVA classification system. SS and ASB, measured using the School Social Behavior Scale (SSBS), were analyzed using fixed-effects models to control for all time-invariant confounders.

**Results:**

Specific UPF subtypes showed differential effects: processed meat was associated with improved SS across all groups, while instant noodles and Western fast food negatively affected SS and positively predicted ASB. Sugar beverage was significantly associated with higher ASB in girls and non-only children, while no significant effect was observed in boys or only child. Snacks and desserts were positively associated with SS in boys and only child but were negatively linked to SS in girls. Fried foods were associated with greater ASB in only children.

**Conclusion:**

UPF consumption is significantly associated with SS deficits and increased ASB among Chinese adolescents, with notable variations by UPF subtype, gender, and only child status. These findings highlight the importance of considering family context and gender in dietary intervention strategies aimed at improving psychosocial outcomes.

## 1 Introduction

Globally, the health issues of adolescents are increasingly garnering attention, with the focus shifting from traditional infectious diseases and malnutrition to mental health and social adaptation issues related to lifestyle and behavior (1–3). Adolescents are maturing in a highly commercialized and digitalized environment, wherein their dietary patterns, habits of daily living, and social behaviors are encountering unprecedented challenges (4, 5). Consequently, investigating the modifiable risk factors that impact their healthy growth is of paramount public health significance for the development of health promotion strategies.

The social development of adolescents is intricately linked to the formation of their social competencies, which pertain to the ability of individuals to confront social tasks and environments positively and effectively (6). The social competencies of adolescents are significantly associated with their social behavioral manifestations (7). Within the school milieu, adolescent social behavior is primarily categorized into social skills (SS) and antisocial behaviors (ASB) (8), both of which serve as pivotal indices for assessing their social competencies.

SS encompass a range of abilities that facilitate effective and appropriate interaction within social contexts (9). They form the core component of social competence, including proficiencies in communication skills, cooperation skills, empathy skills, conflict resolution skills, and the ability to establish and maintain healthy interpersonal relationships (10). From the perspective of developmental psychology, learning SS is a central developmental task during adolescence. Successful development of adolescent can lead to positive social relationships (11), which are not only important protective factors for mental health but also predictive indicators of psychological wellbeing in adulthood (12). Conversely, SS deficits are associated with a series of negative outcomes, including feelings of loneliness and social anxiety (13, 14).

ASB refer to a series of actions that violate social norms, infringe upon the rights of others, or disrupt the normal order of society, ranging from relatively minor acts of defiance, lying, and bullying to severe aggression, violence, and even criminal conduct (15). Among adolescents, ASB is a prevalent and concerning issue, not only causing harm to victims but also posing serious threats to the developmental trajectory of the perpetrators themselves (16), often resulting in academic failure, legal entanglements, and long-term social exclusion (17). Understanding the underlying factors that influence these behaviors is crucial for the prevention and intervention of adolescent ASB.

As defined by the NOVA classification, ultra-processed foods (UPF) are formulations created through a series of industrial processes, incorporating substances beyond sugar, fats, and salt, such as hydrolyzed proteins, modified starches, hydrogenated oils, and additives—including colors, emulsifiers, preservatives, and sweeteners—intended to imitate the sensory qualities of natural foods or to mask undesirable attributes (18). The global prevalence of UPF has surged, with dietary patterns experiencing a shift in food sources, processing methods, and distribution (19). The widespread consumption of these products is driven by their highly palatable nature, low cost, ease of access, and extensive marketing (20, 21). Studies have linked higher intake of UPF to poorer mental health outcomes, including elevated levels of depression, anxiety symptoms, and overall psychological distress (22–24).

High intake of UPF may undermine adolescents' SS and increase the risk of ASB. Preliminary research supports this hypothesis, with studies finding that a nutritious diet is associated with leadership, social-emotional skills, and SS in children (25), and can inversely inhibit aggressive behavior (26). However, research that specifically focuses on the direct association between UPF and the SS and ASB of adolescents remains in its nascent stages, necessitating further in-depth investigation.

When investigating the link between UPF consumption and social behavior, it is essential to account for individual differences, since failing to do so could lead to biased or oversimplified conclusions. Gender and only child status are two crucially modulating variables.

Significant differences exist between boys and girls adolescents. In terms of diet, sex hormones exert varying influences on behavioral inclinations, which may lead to differing sensitivities to physiological changes induced by diet (27, 28). Moreover, gender-based bodily ideals, which often emphasize robust health in boys and slender figures in girls, have become increasingly pronounced and influential in shaping adolescents' dietary behaviors (29). In addition, boys are more prone to exhibit ASB, while girls often demonstrate superior SS and emotional expression, yet they are more susceptible to internalizing behaviors such as depression (17, 30).

It is noteworthy that in countries that have implemented one-child policies, such as China, only child constitute a vast demographic with distinctive socio-psychological characteristics (31). Scholars have posited that only child and non-only child differ in social interactions, with the latter having more time to engage with siblings, while the former enjoy more time spent with parents (32). Only child may have certain advantages in terms of school quality and social environments (33), yet parental overprotection may impede the psychological development of adolescents (34).

Therefore, this study employs a large adolescent sample to systematically examine the relationship between UPF consumption and both SS and ASB, while also assessing the moderating roles of gender and only child status in these associations.

## 2 Methods

### 2.1 Participants

We conducted the survey in accordance with the guiding principles of the Declaration of Helsinki, and all procedures involving human subjects were approved by the Ethics Committee of Shandong University (Approval Number: 20180517). Baseline participants were randomly recruited from secondary schools in 17 cities of Shandong Province using the probability proportional sampling method. The first wave of data was collected in the autumn of 2021, and the second wave was collected in the autumn of 2022 through follow-up surveys. Prior to the survey, informed consent forms were signed by both parents and students. During the data collection process, trained investigators used standardized guidelines to organize students in completing online questionnaires. All data were collected voluntarily, anonymously, and kept confidential.

In 2021, a total of 17,084 samples were collected, and in 2022, 16,494 samples were collected. Firstly, we conducted two rounds of data tracking and matching on the collected samples, retaining 3,705 individuals. Since students who graduated from junior high school and high school at the baseline were already included, the subsequent surveys could no longer track these graduates. Secondly, we excluded missing data such as age, gender, SES, and single-child status, leaving 3,362. Finally, we also excluded UPF, UMF, and quality of life data. After that, a total of 3,206 valid samples were obtained. The samples included 1,510 males (average age 13.62 ± 1.69) and 1,696 females (average age 14.09 ± 1.85).

### 2.2 Measures

#### 2.2.1 Ultra-processed foods

Adolescent participants were instructed to recall and document the variety and consumption frequency of food items they had ingested within the preceding week, adhering to the NOVA Food Classification System. UPF were categorized to include: processed meats, instant noodles, fast food, sweet or savory snacks and desserts, sugary beverages, and fried foods. This classification system has been widely utilized in numerous studies (18).

Food intake frequency was divided into five levels: 0 times per week, 1–2 times per week, 3–5 times per week (every other day), 6–7 times per week (once a day), or eight or more times per week (more than once a day), corresponding to scores of 1–5. Additionally, the six scores were summed to yield a total UPF score ranging from 6 to 30.

#### 2.2.2 Social development

The social development of adolescents was evaluated through the application of the School Social Behavior Scale (SSBS), a tool comprising 65 items that are categorized into two dimensions: SS subscale and the ASB subscale. The SSBS is specifically crafted to gauge the social interactions and performance of adolescents within the school environment, focusing on their relationships with both teachers and peers.

All items are rated on a 5-point Likert scale, with response options ranging from “never” to “often,” corresponding to numerical values of 1–5. Total scores for the SS and ASB scales are computed by summing the ratings of all items in each subscale. Higher scores reflect a greater frequency of the measured behavior. Both scales have demonstrated satisfactory reliability and validity. Specifically, the SS subscale yielded a Cronbach's α of 0.972, KMO = 0.985, and *p* = 0.000; and the ASB subscale showed a Cronbach's α of 0.969, KMO = 0.986, and *p* = 0.000.

#### 2.2.3 Covariates

This study included control variables that might influence the examination outcomes, such as only child status (1 = Yes; 2 = No), gender (1 = Male; 2 = Female), and SES. The SES encompasses parental education level, and self-assessment of the SES is conducted using a 5-point scale (ranging from 1 to 5), with higher scores reflecting a more positive self-perceived family economic situation. The total SES score is derived by summing the scores of all five items.

### 2.3 Statistical analysis

A descriptive analysis was performed using the baseline data collected during the 2021–2023 period. To investigate the disparities in UPF consumption, SS, and ASB based on gender and only child status, chi-square tests and independent samples *t*-tests were applied. Following this, the Hausman test was conducted to ascertain the appropriateness of employing either a fixed effects (FE) or random effects (RE) model for the analysis. The results of the Hausman test led to the rejection of the null hypothesis, suggesting that the FE model was the more appropriate choice for this study. Consequently, a 2-year longitudinal dataset, with the baseline in the autumn of 2021 and the follow-up in the autumn of 2022, was analyzed using an FE model to examine the association between UPF as an explanatory variable and SS and ASB as dependent variables. For the descriptive analysis, SPSS 27.0 (IBM Corp., Armonk, NY, USA) was employed, and Stata 17.0 (Stata Corp LLC, College Station, TX, USA) was utilized for Hausman testing, as well as for assessing robustness, heterogeneity, the variance inflation factor (VIF), and implementing the FE model.

To address unobserved heterogeneity at the individual level, longitudinal estimation and heterogeneity analysis were conducted using data from both waves of the study. The robustness of the findings was tested by verifying the robustness of the *t*-statistics to ensure the stability of the results. Heterogeneity was explored by examining differences in outcomes by gender and by the type of UPF consumed. To assess the presence of multicollinearity among the study variables, VIF calculations were carried out. Additionally, the FE model utilized in this research helped to mitigate potential endogeneity concerns.

## 3 Results

### 3.1 Descriptive analysis

Table 1 describes the distribution of baseline characteristics for both boys and girls. The total sample size of this study was 3,206, with 1,510 boys (47.10%) and 1,696 girls (52.90%). Among them, 1,867 were non-only children (58.23%), and 1,339 were only child (41.77%). In terms of educational background within the SES framework, the predominant level achieved by fathers is “junior high school” (34.53%), followed by “senior high school” (17.47%) and “vocational high school” (11.76%). Mothers' educational qualifications are largely concentrated in “junior high school” (35.40%), with “senior high school” (15.22%) being the next most common level.

**Table 1:** Characteristics of study participants (*n* = 3,206).

Item	Characteristics	Frequency	Percentage (%)	Cumulative percentage (%)
Gender	Boys	15,10	47.10	47.10
	Girls	1,696	52.90	100.00
Only child status	Only child	1,339	41.77	41.77
	Non-only children	1,867	58.23	100.00
Father's education	Uneducated	57	1.78	1.78
	Primary school	207	6.46	8.23
	Junior high school	1,107	34.53	42.76
	Technical school	377	11.76	54.52
	Vocational high school	180	5.61	60.14
	Senior high school	560	17.47	77.60
	Junior college	359	11.20	88.80
	Undergraduate	251	7.83	96.63
	Postgraduate and above	108	3.37	100.00
Mother's education	Uneducated	73	2.28	2.28
	Primary school	337	10.51	12.79
	Junior high school	1,135	35.40	48.19
	Technical school	344	10.73	58.92
	Vocational high school	151	4.71	63.63
	Senior high school	488	15.22	78.85
	Junior college	336	10.48	89.33
	Undergraduate	257	8.02	97.35
	Postgraduate and above	85	2.65	100.00
Total		3,206	100.0	100.0

As shown in Appendix Table A1, the occupational profiles of fathers are predominantly characterized by “farmers” (14.50%), “self-employed individuals” (15.44%), and “skilled workers” (13.23%), while the occupational distribution for mothers is headed by “farmers” (17.59%), “self-employed individuals” (16.19%), and “skilled workers” (10.29%).

### 3.2 Ultra-processed foods, social skills, and antisocial behavior

As illustrated in Table 2, gender differences in UPF, SES, SS, and ASB were analyzed. Boys exhibited a higher frequency of processed meat consumption than girls. Similarly, a greater proportion of boys consumed instant noodles across all frequency categories, whereas approximately 37% of girls reported no consumption. For Western fast food, boys also showed higher consumption frequencies, with 37% of girls reporting non-consumption. In contrast, girls had a higher frequency of sugary beverage consumption at 6–7 times/week and ≥8 times/week, while boys had higher proportions at lower frequencies (1–2 and 3–5 times/week). Regarding snacks and desserts, a significantly larger proportion of boys reported no consumption, whereas girls more frequently consumed these items 1–2 times/week and 6–7 times/week. Fried food consumption frequencies were similar between sexes, though a slightly higher proportion of boys reported consumption ≥8 times/week compared to girls.

**Table 2:** Gender differences among participants.

Characteristics	Gender (%)		χ^2/^*t*	*p*-Value
	Boys	Girls		
Processed meats			52.339	0.000^**^
0 times per week	147 (9.74)	166 (9.79)		
1–2 times per week	441 (29.21)	667 (39.33)		
3–5 times per week	447 (29.60)	492 (29.01)		
6–7 times per week	292 (19.34)	217 (12.79)		
≥8 times per week	183 (12.12)	154 (9.08)		
Instant noodles			71.507	0.000^**^
0 times per week	385 (25.50)	627 (36.97)		
1–2 times per week	677 (44.83)	689 (40.63)		
3–5 times per week	211 (13.97)	208 (12.26)		
6–7 times per week	126 (8.34)	123 (7.25)		
≥8 times per week	111 (7.35)	49 (2.89)		
Western fast food			61.231	0.000^**^
0 times per week	551 (36.49)	740 (43.63)		
1–2 times per week	571 (37.81)	706 (41.63)		
3–5 times per week	186 (12.32)	121 (7.13)		
6–7 times per week	102 (6.75)	66 (3.89)		
≥8 times per week	100 (6.62)	63 (3.71)		
Sugary beverages			39.303	0.000^**^
0 times per week	448 (29.67)	481 (28.36)		
1–2 times per week	628 (41.59)	803 (47.35)		
3–5 times per week	193 (12.78)	247 (14.56)		
6–7 times per week	110 (7.28)	97 (5.72)		
≥8 times per week	131 (8.68)	68 (4.01)		
Snacks and desserts			55.680	0.000^**^
0 times per week	401 (26.56)	308 (18.16)		
1–2 times per week	548 (36.29)	781 (46.05)		
3–5 times per week	324 (21.46)	361 (21.29)		
6–7 times per week	131 (8.68)	172 (10.14)		
≥8 times per week	106 (7.02)	74 (4.36)		
Fried foods			9.593	0.048^*^
0 times per week	295 (19.54)	333 (19.63)		
1–2 times per week	763 (50.53)	876 (51.65)		
3–5 times per week	277 (18.34)	306 (18.04)		
6–7 times per week	80 (5.30)	111 (6.54)		
≥8 times per week	95 (6.29)	70 (4.13)		
SES	22.22 ± 8.87	22.86 ± 8.37	−2.088	0.037^*^
Age	13.62 ± 1.69	14.09 ± 1.85	−7.641	0.000^**^
SS	119.74 ± 28.02	122.14 ± 24.80	−2.548	0.011^*^
ASB	48.76 ± 19.89	43.32 ± 14.95	8.661	0.000^**^

SES, socioeconomic status; ASB, antisocial behavior; SS, social skills.

^**^p < 0.01, ^*^p < 0.05.

Additionally, significant gender differences were found in SES, SS, and ASB. Girls had a higher SES (22.86 ± 8.37) compared to boys (22.22 ± 8.87), girls had higher SS scores (122.14 ± 24.80) compared to boys (119.74 ± 28.02), and boys had higher ASB scores (48.76 ± 19.89) compared to girls (43.32 ± 14.95).

**Figure 1:**
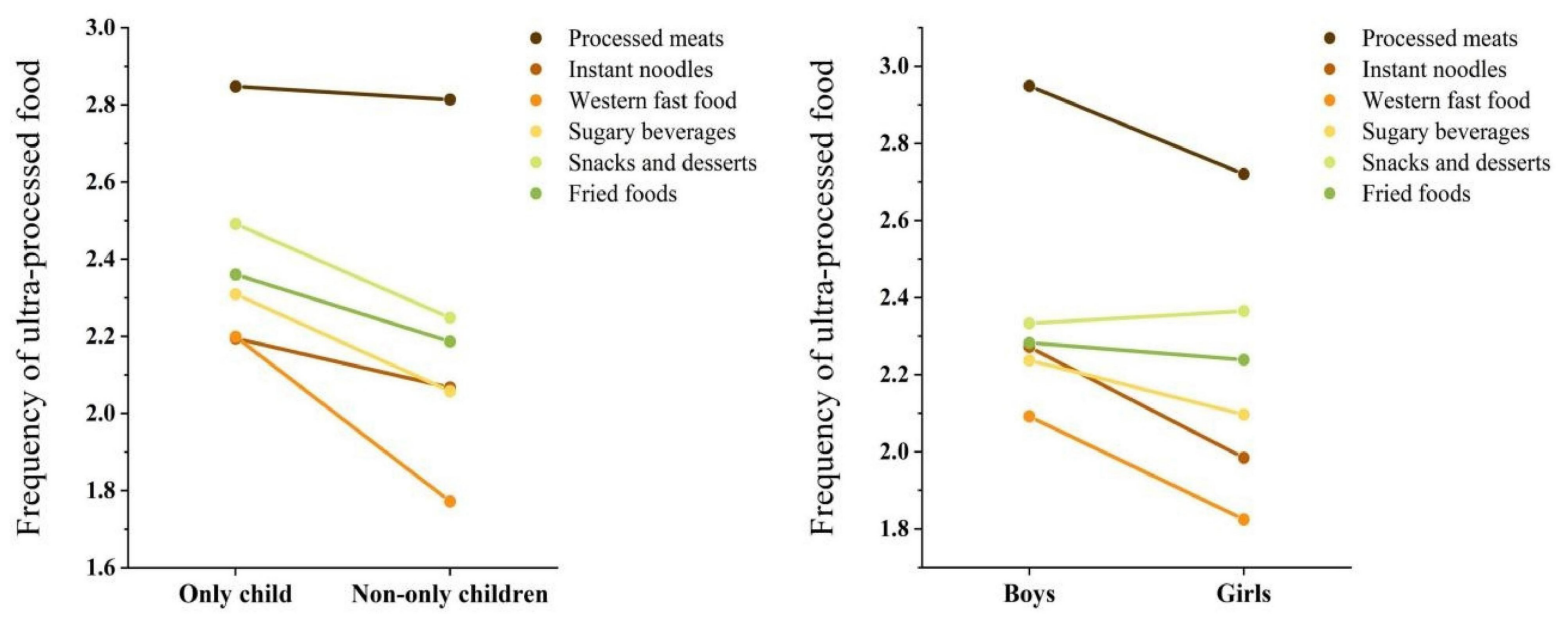
Differences in ultra-processed foods by gender and only-child status.

As presented in Table 3, differences in intake frequency of UPF, beverages, and snacks/desserts were examined by only-child status. Only children had a higher proportion of processed meat consumption. For instant noodles, only children demonstrated markedly higher consumption frequencies at 1–2, 6–7, and ≥8 times/week. Similarly, a higher proportion of only children consumed Western fast food at frequencies of 1–2, 6–7, and ≥8 times/week; notably, 84.42% of non-only children reported consumption at 0 or 1–2 times/week. Regarding sugary beverages, only children exhibited substantially higher intake at 6–7 and ≥8 times/week, while non-only children had a significantly higher proportion of non-consumption. For snacks and desserts, only children showed elevated consumption at 3–5 and ≥8 times/week, whereas non-only children were more likely to report no consumption. Fried food intake followed a similar pattern, with only children consuming more frequently at 6–7 and ≥8 times/week, and non-only children more often reporting no consumption. These differences by gender and only-child status are visualized in Figure 1.

**Table 3:** Differences among participants by only child status.

Characteristics	Only child (%)		*χ^2/^t*	*p*-Value
	Only child	Non-only children		
Processed meats			20.359	0.000^**^
0 times per week	154 (11.50)	159 (8.52)		
1–2 times per week	420 (31.37)	688 (36.85)		
3–5 times per week	383 (28.60)	556 (29.78)		
6–7 times per week	240 (17.92)	269 (14.41)		
≥8 times per week	142 (10.60)	195 (10.44)		
Instant noodles			66.176	0.000^**^
0 times per week	395 (29.50)	617 (33.05)		
1–2 times per week	600 (44.81)	766 (41.03)		
3–5 times per week	119 (8.89)	300 (16.07)		
6–7 times per week	139 (10.38)	110 (5.89)		
≥8 times per week	86 (6.42)	74 (3.96)		
Western fast food			156.000	0.000^**^
0 times per week	402 (30.02)	889 (47.62)		
1–2 times per week	590 (44.06)	687 (36.80)		
3–5 times per week	127 (9.48)	180 (9.64)		
6–7 times per week	119 (8.89)	49 (2.62)		
≥8 times per week	101 (7.54)	62 (3.32)		
Sugary beverages			53.106	0.000^**^
0 times per week	328 (24.50)	601 (32.19)		
1–2 times per week	608 (45.41)	823 (44.08)		
3–5 times per week	174 (12.99)	266 (14.25)		
6–7 times per week	119 (8.89)	88 (4.71)		
≥8 times per week	110 (8.22)	89 (4.77)		
Snacks and desserts			75.918	0.000^**^
0 times per week	217 (16.21)	492 (26.35)		
1–2 times per week	565 (42.20)	764 (40.92)		
3–5 times per week	342 (25.54)	343 (18.37)		
6–7 times per week	112 (8.36)	191 (10.23)		
≥8 times per week	103 (7.69)	77 (4.12)		
Fried foods			31.958	0.000^**^
0 times per week	238 (17.77)	390 (20.89)		
1–2 times per week	669 (49.96)	970 (51.96)		
3–5 times per week	235 (17.55)	348 (18.64)		
6–7 times per week	105 (7.84)	86 (4.61)		
≥8 times per week	92 (6.87)	73 (3.91)		
SES	25.10 ± 8.93	20.73 ± 7.89	14.334	0.000^**^
Age	13.76 ± 1.95	13.95 ± 1.66	−2.898	0.004^**^
SS	122.01 ± 27.35	120.29 ± 25.65	1.802	0.072
ASB	48.39 ± 20.72	44.08 ± 14.83	6.514	0.000^**^

SES, socioeconomic status; ASB, antisocial behavior; SS, social skills.

^**^p < 0.01, ^*^p < 0.05.

Additionally, significant differences in SES, SS, and ASB were found based on only child status. The only child had a significantly higher SES (25.10 ± 8.93), SS (122.01 ± 27.35), and ASB (48.76 ± 19.89).

### 3.3 The relationship between ultra-processed and social skills and antisocial behavior

#### 3.3.1 Correlation analysis

As shown in Table 4, there was a significant negative correlation between UPF and SS (*r* = −0.296, *p* < 0.01), and a significant positive correlation between UPF and ASB (*r* = 0.425, *p* < 0.01). There was a positive correlation between SES and UPF (*r* = 0.036, *p* < 0.05), and a significant correlation between SES and SS (*r* = 0.099, *p* < 0.01). Age has a significant positive correlation with UPF (*r* = 0.141, *p* < 0.01), a significant negative correlation with SS (*r* = −0.126, *p* < 0.01), and a significant positive correlation with ASB (*r* = 0.076, *p* < 0.01).

**Table 4:** Correlation analysis.

Item	Mean	SE	Age	SES	UPF	SS	ASB
Age	13.869	1.790	1				
SES	22.557	8.612	−0.001	1			
UPF	13.671	5.064	0.141^**^	0.036^*^	1		
SS	121.009	26.386	−0.126^**^	0.099^**^	−0.296^**^	1	
ASB	45.882	17.659	0.076^**^	0.030	0.425^**^	−0.477^**^	1

SES, socioeconomic status; ASB, antisocial behavior; SS, social skills; UPF, ultra-processed foods.

^*^p < 0.05; ^**^p < 0.010.

#### 3.3.2 Impact of ultra-processed foods on social skills and differences by gender and only child status

As shown in Table 5, after controlling for age and SES, the data indicated that the ingestion of processed meats was positively correlated with SS in adolescents (All: β = 1.849, *t* = 4.31). Consumption of instant noodles was found to have a statistically significant negative impact on the SS of all adolescent participants (All: β = −3.389, *t* = −6.11). Western fast food was identified to have a statistically significant adverse effect on the SS of all participants (All: β = −2.641, *t* = −4.58). Snack and desserts had a divergent impact on SS, exerting a positive effect for boys (β = 1.823, *t* = 2.17) and a negative effect for girls (β = −1.122, *t* = −1.66). Simultaneously, snack and desserts had a significant positive effect on the SS of only child (β = 2.082, *t* = 2.34). Fried foods exerted a significant detrimental effect on SS of the entire adolescent cohort (All: β = −3.437, *t* = −5.69).

**Table 5:** Fixed effects model results of ultra-processed foods on social skills.

Variables	All	Boys	Girls	Only child	Non-only children
Processed meats	1.849^***^	1.521^**^	2.278^***^	1.525^**^	2.201^***^
	(4.31)	(2.36)	(4.04)	(2.30)	(3.97)
Instant noodles	−3.389^***^	−3.120^***^	−4.145^***^	−5.174^***^	−2.221^***^
	(−6.11)	(−3.61)	(−5.71)	(−5.51)	(−3.31)
Western fast food	−2.641^***^	−3.751^***^	−1.826^**^	−2.733^***^	−2.680^***^
	(−4.58)	(−4.26)	(−2.39)	(−2.86)	(−3.76)
Sugary beverages	−2.044^***^	−1.146	−2.781^***^	−1.843^*^	−2.190^***^
	(−3.36)	(−1.18)	(−3.76)	(−1.79)	(−3.00)
Snacks and desserts	0.279	1.823^**^	−1.122^*^	2.082^**^	−0.940
	(0.52)	(2.17)	(−1.66)	(2.34)	(−1.45)
Fried foods	−3.437^***^	−4.224^***^	−2.365^***^	−4.954^***^	−2.134^***^
	(−5.69)	(−4.48)	(−3.15)	(−5.25)	(−2.81)
Age	−0.197	−0.327	−0.079	−2.109^**^	1.315^*^
	(−0.35)	(−0.37)	(−0.11)	(−2.40)	(1.74)
SES	0.270^***^	0.096	0.462^***^	0.276^***^	0.245^***^
	(4.68)	(1.10)	(6.29)	(3.34)	(3.06)
Constant	136.296^***^	140.469^***^	131.830^***^	167.882^***^	111.310^***^
	(16.44)	(11.36)	(11.88)	(13.08)	(10.12)
*R* ^2^	0.129	0.120	0.156	0.185	0.102
Adj. *R*^2^	0.128	0.117	0.154	0.183	0.100
*F*	42.22	18.97	28.72	30.68	17.92

All models controlled for age and SES.

SES, socioeconomic status.

^***^p < 0.01; ^**^p < 0.05, ^*^p < 0.1.

Robust t-statistics in parentheses.

#### 3.3.3 Impact of ultra-processed foods on antisocial behavior and differences by gender and only child status

As illustrated in Table 6, after controlling for age and SES, although processed meats were significantly associated with lower ASB among boys, girls, and non-only children (Boy: β = −0.760, *t* = −1.75; Girl: β = −0.944, *t* = −2.48; Only child: β = −0.784, *t* = −1.58; Non-only children: β = −0.991, *t* = −2.87). Instant noodles were significantly associated with higher ASB in all adolescents (All: β = 1.993, *t* = 5.03). Western fast food was significantly associated with higher ASB in all participants (All: β = 2.916). Sugary beverages were significantly associated with higher ASB in girls and non-only children (Girl: β = 1.367, *t* = 2.38; Non-only children: β = 1.330, *t* = 2.47). Notably, snack foods and desserts did not have a significant impact on ASB in all participants. Fried food consumption significantly increased ASB among boys, girls, and only children (Boy: β = 2.293, *t* = 2.80; Girl: β = 1.308, *t* = 2.43; Only child: β = 3.752, *t* = 4.51).

**Table 6:** Fixed effects model results of ultra-processed foods on antisocial behavior.

Variables	All	Boys	Girls	Only child	Non-only children
Processed meats	−0.865^***^	−0.760^*^	−0.944^**^	−0.784	−0.991^***^
	(−3.00)	(−1.75)	(−2.48)	(−1.58)	(−2.87)
Instant noodles	1.993^***^	1.528^**^	2.479^***^	3.516^***^	1.063^**^
	(5.03)	(2.37)	(5.13)	(5.05)	(2.32)
Western fast food	2.916^***^	3.470^***^	2.383^***^	2.249^***^	3.282^***^
	(6.34)	(4.64)	(4.14)	(3.11)	(5.57)
Sugary beverages	1.002^**^	0.601	1.367^**^	0.418	1.330^**^
	(2.21)	(0.85)	(2.38)	(0.54)	(2.47)
Snacks and desserts	0.098	−0.523	0.796	0.456	−0.078
	(0.24)	(−0.80)	(1.52)	(0.63)	(−0.16)
Fried foods	1.820^***^	2.293^***^	1.308^**^	3.752^***^	0.155
	(3.72)	(2.80)	(2.43)	(4.51)	(0.28)
Age	−0.288	−1.163^*^	0.501	−0.950	0.275
	(−0.72)	(−1.81)	(1.05)	(−1.41)	(0.57)
SES	0.160^***^	0.186^***^	0.132^**^	0.192^***^	0.107^*^
	(3.73)	(2.71)	(2.57)	(2.87)	(1.93)
Constant	32.301^***^	46.621^***^	18.863^***^	35.274^***^	29.901^***^
	(5.60)	(5.20)	(2.59)	(3.60)	(4.21)
*R* ^2^	0.149	0.128	0.186	0.217	0.109
Adj. *R*^2^	0.148	0.125	0.184	0.214	0.107
*F*	42.49	17.79	29.01	31.63	16.08

All models controlled for age and SES.

SES, socioeconomic status.

^***^p < 0.01; ^**^p < 0.05; ^*^p < 0.1.

Robust t-statistics in parentheses.

## 4 Discussion

The study's findings reveal significant gender and only child status differences in the frequency of UPF intake. Boys reported significantly higher consumption of processed meats, instant noodles, Western fast food, sugary beverages, and fried foods relative to girls, which aligns with previous research (35). Studies suggest that due to hormonal influences, menstrual cycles, or personal preferences, girls are more inclined toward foods such as vegetable salads, fruits, and desserts (36, 37). Additionally, girls are more sensitive to self-weight and appearance satisfaction and may resort to dietary control to achieve a desired body shape (38).

Furthermore, the results indicate that only children reported significantly higher consumption of instant noodles, Western fast food, sugary beverages, snacks and desserts, and fried foods than non-only children. A plausible explanation is that only child, due to receiving more attention and less parental supervision at home, may consume more UPF (39, 40). Moreover, parents might even use UPF as rewards for their only children (41).

Consistent with prior studies, we found that the processed meat consumption was significantly positively associated with SS in all adolescents (42). From a social-eating perspective, processed meats are frequently consumed in communal settings such as barbecues, hot-spot meals, and shared dinners that inherently facilitate peer interaction and group cohesion (43). Nevertheless, reverse causality that adolescents with better social skills may be more likely to participate in such social eating occasions and residual confounding by extroversion or socioeconomic context cannot be ruled out. Conversely, instant noodles, Western fast food, and fried foods had a detrimental effect on adolescents' SS. This could be attributed to the increased prevalence of mental health issues among adolescents due to excessive UPF intake (44), which is closely linked to the absence of SS (45), leading to a decline in SS. Additionally, the obesity and overweight associated with UPF consumption may also impact SS (46).

Sugary beverages demonstrated a significant negative effect on SS in girls, although no significant effect was observed in boys. Additionally, research has established a positive association between sugary beverage consumption and obesity (47), Compared to boys, girls are more sensitive to appearance and weight (38), and concerns about appearance can cause psychological distress (48, 49), potentially affecting SS.

Snack and desserts were positively associated with SS in boys and only children, but to have a negative impact on the SS of girls. Some scholars suggest that food can serve as a social lubricant, particularly for easily shareable items such as snacks and desserts (50). Snack and desserts are easily shareable and frequently exchanged in adolescent peer groups, which signals trust, promotes cooperation, and strengthens in-group bonds (50). Boys, who are adept at group interactions (51), may gain a sense of self-identity through sharing food, thereby promoting their SS (43, 50). In contrast, girls tend to prefer emotional exchanges (51), and thus snacks and desserts do not exert a positive influence on their SS. For only children, who lack sibling interaction, sharing snacks may serve as a deliberate strategy to initiate friendships and compensate for reduced social practice at home, thereby accelerating SS development.

As predicted, the act of sharing food among only child was found to be conducive to the enhancement of their SS. Within the context of China's one-child policy, only children have been found to demonstrate positive social competencies (52). A potential reason for this is that the absence of siblings may limit the opportunities for only child to engage in social interactions with peers, prompting them to seek out social interactions with peers outside the family and to foster their SS through the sharing of snacks or desserts to compensate for the lack of social interactions within the family.

Instant noodles and Western fast food were found to have a significant impact on ASB in all adolescents. Excessive intake of instant noodles has been linked to sleep disturbances, such as shortened sleep duration (53) and poor sleep quality (54), which is closely related to ASB of adolescent (55). Western fast food is one of the main causes of adolescent depression (56), which can lead to severe psychological distress (57). Scholars have confirmed that psychological distress can exacerbate ASB such as bullying and criminal activity in adolescents (58, 59).

Interestingly, sugary beverages were positively associated with ASB in girls and non-only children, but had no effect on boys and only child. A potential explanation for this is that sugary beverages can contribute to adolescent obesity (57). Girls, more prone to appearance anxiety and weight sensitivity than boys (38), may therefore experience an increase in ASB due to sugary beverages.

The finding that sugary beverages were associated with higher ASB in non-only child may be attributed to factors such as lower parental attention and family economic status within the home environment. To our knowledge, other scholars have not yet examined the differential impact of only child status on the association between sugary beverages and ASB. Future research should delve deeper into this unique Chinese demographic to further understand the underlying mechanisms of these differences.

### 4.1 Limitations

Several limitations should be considered. First, the dietary assessment relied on a self-reported food frequency questionnaire that captured only the intake frequency of six broad UPF categories without portion sizes, introducing recall bias and measurement error. Second, SS and ASB were also self-reported via the SSBS, which may be subject to social desirability bias. Third, despite using a two-wave longitudinal design and FE to control for time-invariant confounders, the observational nature of the study precludes causal inference. Fourth, residual confounding remains a concern: we adjusted for age and SES, but unmeasured factors, such as physical activity, BMI, sleep quality, mental health status, parenting style, screen time, and peer influence, could affect both UPF intake and social development. Fifth, the sample was drawn from China, where the one-child policy has created a unique family structure, limiting generalizability to other cultural or socioeconomic contexts. These limitations highlight the need for more comprehensive dietary measures, broader confounder assessment, and more diverse populations in future research.

## 5 Conclusion

This study explored the relationship between UPF intake and SS and ASB in Chinese adolescents, revealing differences by gender and only child status. The findings indicate that higher total UPF intake is associated with poorer SS and increased ASB, but the impact of different UPF varies. For instance, processed meats have a positive effect on SS, while instant noodles and Western fast food generally have a negative impact. The effect of sugary beverages on ASB is more pronounced in girls and non-only children, and snacks and desserts were positively associated with SS in boys and only children but negatively associated with SS in girls. The results suggest that the influence of UPF on adolescent social development is modulated by the type of food, gender, and only child status. Therefore, it is advisable that future nutritional interventions be tailored to the specific demographic and behavioral characteristics of different adolescent groups to enhance their social adaptability effectively.

## Data Availability

The datasets presented in this study can be found in online repositories. The names of the repository/repositories and accession number(s) can be found below: Data can be requested and downloaded through the website: https://www.ncmi.cn//phda/dataDetails.do?id=CSTR:17970.11.A0031.202107.209.V1.0.

## Appendix

**Table A1:** Parents' job of study participants (*n* = 3,206).

Item	Characteristics	Frequency	Percentage (%)	Cumulative percentage (%)
Father's job	Unemployed and laid-off workers	345	10.76	10.76
	Farmers	465	14.50	25.27
	Self-employed	495	15.44	40.70
	General employees in commerce and services	86	2.68	43.39
	General workers	309	9.64	53.03
	Skilled workers	424	13.23	66.25
	Private business owners	122	3.81	70.06
	General clerks and assistants	311	9.70	79.76
	Technicians	105	3.28	83.03
	Professionals such as teachers, engineers, doctors, and lawyers	158	4.93	87.96
	Senior management in enterprises or companies	234	7.30	95.26
	Leaders or department heads in government agencies or public institutions	152	4.74	100.00
Mother's job	Unemployed and laid-off workers	328	10.23	10.23
	Farmers	564	17.59	27.82
	Self-employed	519	16.19	44.01
	General employees in commerce and services	303	9.45	53.46
	General workers	281	8.76	62.23
	Skilled workers	330	10.29	72.52
	Private business owners	102	3.18	75.70
	General clerks and assistants	246	7.67	83.37
	Technicians	52	1.62	85.00
	Professionals such as teachers, engineers, doctors, and lawyers	317	9.89	94.88
	Senior management in enterprises or companies	110	3.43	98.32
	Leaders or department heads in government agencies or public institutions	54	1.68	100.00
Total		3,206	100.0	100.0
